# Retraction Note: Overexpression of miR-499-5p inhibits non-small cell lung cancer proliferation and metastasis by targeting VAV3

**DOI:** 10.1038/s41598-021-96814-6

**Published:** 2021-08-23

**Authors:** Ming Li, Shenjun Zhang, Ning Wu, Liang Wu, Changhui Wang, Yinping Lin

**Affiliations:** 1 Department of Respiratory Medicine, Shanghai 10th People’s Hospital, Tongji University, Shanghai, 200072 China; 2 Institute of Biochemistry and Biology, Shanghai Institutes for Biological Sciences, Chinese Academy of Sciences, Shanghai, 200031 China; 3 Department of Thoracic Surgery, Huashan Hospital, Fudan University, Shanghai, 200032 China; 4 Department of Thoracic Surgery, Shanghai Pulmonary Hospital, Tongji University, Shanghai, 200433 China

Retraction of: *Scientific Reports* 10.1038/srep23100, published online 14 March 2016

The Editors have retracted this Article.

Several of the figures presented in this paper appear to contain duplications that are not compliant with the digital image and standards policy of the journal, specifically:

- There appears to be partial overlap of the actin panels presented in Figure 2A
- There appears to be a high level of similarity between the actin bands presented in Figure 3A
- There appears to be duplication within the panels presented in Figure 3C
- There appears to be duplication within and between the panels presented in Figure 3D
- There appears to be duplication of the panels for β-actin in Figure 4C

In addition, the last author has stated they were not able to locate all the original data files. The Editors therefore no longer have confidence in the reliability of the data reported in the Article.

The authors have not responded to correspondence regarding this retraction.

